# Circulating GRP78 antibodies from ovarian cancer patients: a promising tool for cancer cell targeting drug delivery system?

**DOI:** 10.18632/oncotarget.22412

**Published:** 2017-11-11

**Authors:** Kylie Van Hoesen, Sonia Meynier, Pascale Ribaux, Patrick Petignat, Florence Delie, Marie Cohen

**Affiliations:** ^1^ Department of Gynecology Obstetrics, University of Geneva, 1205 Geneva, Switzerland; ^2^ School of Pharmaceutical Sciences, University of Geneva, University of Lausanne, 1205 Geneva, Switzerland

**Keywords:** membrane GRP78, anti-GRP78 autoantibodies, targeted therapy, ovarian cancer, chorioallantoic membrane

## Abstract

Glucose-regulated protein 78 (GRP78) is a chaperone protein that has a high frequency in tumor cells. Normally it is found in the endoplasmic reticulum to assist in protein folding, but under cellular stress, GRP78 influences proliferative signaling pathways at the cell surface. The increased expression elicits autoantibody production, providing a biomarker of ovarian cancer, as well as other types of cancer. This study aims to determine the epitope recognition of GRP78 autoantibodies isolated from serum of ovarian cancer patients and use the identified antibodies to design new drug delivery systems to specifically target cancer cells. We first confirmed that the membrane GRP78 levels are increased in ovarian cancer cells and positively correlate with proliferation. However, the level of circulating GRP78 autoantibodies did not correlate with membrane GRP78 expression in ovarian cancer cells and was lower, although not significantly, compared to control patients. We then determined the epitope recognition of GRP78 autoantibodies and showed that treatment with paclitaxel-loaded nanoparticles coated with anti-GRP78 antibodies significantly decreased tumor development in chick embryo culture of ovarian cancer cell tumors compared to paclitaxel treatment alone. This evidence suggests that nanoparticle drug delivery systems coupled with antibodies against GRP78 has potential as a powerful therapy against ovarian cancer.

## INTRODUCTION

The risk of women developing invasive ovarian cancer is 1 in 75, making it the seventh most common cancer in women worldwide. Ovarian cancer ranks fifth in cancer-related deaths in the United States among women. Currently, about 45% of patients survive five years. Poor survival rates are associated with late stage diagnoses and high rates of recurrence. Current treatment includes surgery and chemotherapy [1–3]. Late stage prognosis is due to the lack of effective diagnostic methods, with only 23% of ovarian cancers detected at stage I. For some types of cancer, current detection is facilitated with antigen-based diagnostic assays, which detects the overexpression of specific serum proteins. Cancer specificity is amplified by inducing autoantibody responses against tumor-derived proteins, which can be identified before the detection of circulating antigens [4].

Glucose-regulated protein 78 (GRP78) is a chaperone protein that assists in protein folding and is conventionally located in the lumen of the endoplasmic reticulum (ER) where it works to regulate ER homeostasis [5, 6]. Typically, adult tissues have low levels of GRP78, but the expression varies based on tissue type and developmental stage. The expression of GRP78 is induced under conditions of stress, including hypoxia and nutrient deprivation, and is observed at high levels in tumor cells [6–8]. Under stress, GRP78 can exhibit different functions at various locations in the cell to aid in the survival of the cell, including increasing cell proliferation and decreasing apoptosis. It has been demonstrated that tumor progression is hindered by reducing GRP78 levels in heterozygous mice [9].

In various types of cancer cells, including lung and colon adenocarcinomas, neuroblastoma, and ovarian tumors, GRP78 is found at the cell surface [10] where it can influence signaling pathways by binding to other proteins, leading to the proliferation and invasion of tumors [6]. For example, GRP78 at the cell surface interacts with α2-macroglobulin (α_2_M) to incite proliferation of human prostate cancer cells [11]. The cell surface expression of GRP78 in prostate cancer correlates with the metastatic behavior observed in mice [12]. GRP78 has also been shown to modulate tissue factor (TF) procoagulant activity (PCA), which is known to contribute to venous thromboembolism in cancer patients, leading to disability or death. TF is also associated with cancer progression and metastasis. Cell surface GRP78 correlates with the expression of TF/PCA during anti-GRP78 autoantibody engagement with cell surface GRP78. Decreasing GRP78 cell surface signaling efficiently decreases TF/PCA activity and reduces cancer-related thrombosis as well as decreasing tumor growth [13]. GRP78 can also be found in the cytoplasm, where it blocks apoptosis pathways [14].

When GRP78 is relocated to the cell surface, the increased expression and localization initiates an immune response, resulting in the production of autoantibodies [15], which can serve as a biomarker to help with cancer detection in ovarian cancer [6]. Initially, Mintz et al. reported that autoantibodies were present only in patients with prostate cancer, but then Taylor et al revealed antibodies against GRP78 in the sera of ovarian cancer patients [5, 16]. In addition to diagnostic properties, autoantibodies have shown to influence the progression of tumors in a murine melanoma model [11]. Patients with seric autoantibodies against GRP78 indicate predictions of shorter overall survival in prostate cancer patients. [16] Deeper investigation into the epitope function of anti-GRP78 antibodies in prostate cancer showed that the antibodies bind and induce proliferation of tumor cells that have GRP78 at the surface. The GRP78 primary amino acid sequence (Leu98-Leu115) is assumed to bind to activated α_2_M [12]. However, for prostate and melanoma cell lines, commercial antibodies against the C-terminal domain of GRP78 inhibit cellular proliferation and promote apoptosis by acting as receptor antagonists, blocking the activation of GRP78 and also upregulating p53 (tumor suppressor gene) [17]. Whereas, antibodies purified from prostate cancer patients appear to induce cell proliferation [11–13], GRP78 antibodies purified from ovarian cancer patients increase apoptosis and decrease invasive characteristics [15]. The contrast in antibody effects may depend on the GRP78 epitope. Determining the epitope recognized by GRP78 autoantibodies isolated from sera is necessary to effectively utilize antibody therapy for targeted treatment.

Since GRP78 is specifically expressed at the surface of cancer cells, it can act as a target for antibodies against the C-terminus of the protein [6]. Targeted therapy for ovarian cancer is used to pinpoint GRP78 in cancer cells, while reducing adverse effects in healthy tissues. One approach to target GRP78 is nanoparticles (NPs). Nanoparticles are beneficial for poorly-water soluble drugs, such as paclitaxel (Ptx), because the drugs are then delivered in suspension rather than solubilized in toxic excipient such as Cremophor EL, responsible for side effects. Paclitaxel, commercialized as Taxol^®^ is a common drug used in treatment against cancer inducing cell division arrest at the G2 mitotic stage, and inhibits angiogenesis and cell migration [18]. NPs are made of polymers to support longer circulation and enhanced permeability. Also, NPs can be surface coupled with monoclonal antibodies to target specific cells [19]. This aspect of nanomedicine is attractive because it reduces the side effects on healthy cells since NPs can specifically target cancer cells.

In this study, we quantified GRP78 autoantibodies in serum of patients with ovarian benign disease and high grade serous ovarian cancer (HGSOC) to determine if they can be used as biomarkers of HGSOC. We then examined the GRP78 epitopes recognized by GRP78 autoantibodies from ascites and serum of the same HGSOC patient, and determined which epitope could be involved in pro-apoptotic activity. Finally, Ptx loaded NPs were coupled with GRP78 antibodies recognizing this epitope to explore the cytotoxic effects of the treatment on ovarian cancer cells *in vitro* and *in vivo* using chick embryo culture.

## RESULTS

### Membrane GRP78 expression in ovarian benign and cancer cells

**Figure 1:**
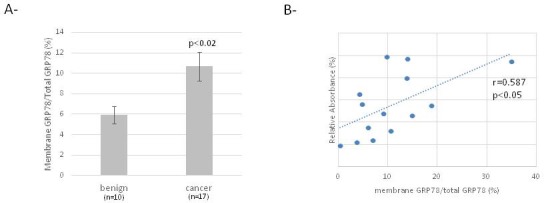
Membrane GRP78 expression in ovarian benign and cancer cells **(A)** Cell-ELISA for detection of membrane and total expression of GRP78 in primary ovarian cells. **(B)** Correlation curve between relative membrane expression of GRP78 and relative cell proliferation.

We observed the presence of GRP78 at the cell surface of primary ovarian benign cells and cancer cells (Figure 1A). Nevertheless, the level of membrane GRP78 is increased in ovarian cancer cells compared to benign cells and seems to correlate with the proliferative capacity of the cells (Figure 1B).

### Circulating GRP78 autoantibodies levels

**Figure 2:**
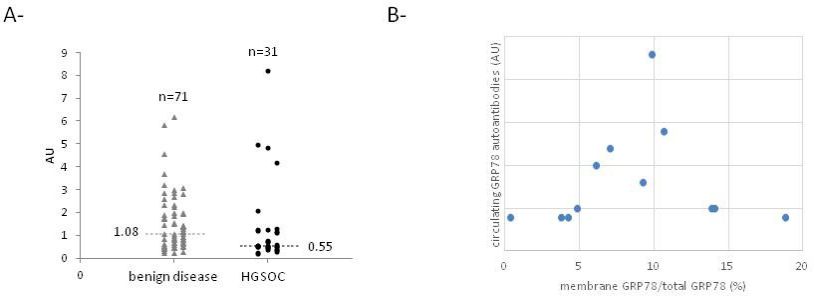
GRP78 autoantibodies level in serum of control and HGSOC patients **(A)** Level of circulating anti-GRP78 antibodies in serum of control and HGSOC patients determined by ELISA. **(B)** Correlation curve between relative GRP78 membrane expression in ovarian cells and circulating level of anti-GRP78 autoantibodies.

GRP78 autoantibodies were quantified in serum of control (n=71) and HGSOC (n=31) patients by ELISA. Despite a lower mean level of GRP78 autoantibodies in serum of ovarian cancer patients compared to controls, this difference is not statistically significant (Figure 2A). Moreover, the level of these antibodies did not correlate with the membrane GRP78 level in ovarian cells (Figure 2B).

### Epitope mapping

GRP78 epitope mapping of ascites and serum was acquired through translation of a protein sequence of GRP78 into 13mer peptides with an amino acid shift, and elongating the C- and N-termini. Several different epitopes were recognized, signifying the presence of more than one GRP78 autoantibody in serum and ascites (Table 1 and Supplementary Data). Since we previously found that GRP78 autoantibodies purified from ascites and serum exhibit different properties on cells, we then identified some peptides that may react differently in serum compared to ascites (Table 2).

**Table 1:** Main GRP78 epitopes recognized by GRP78 autoantibodies from ascites and serum of HGSOC patients

Main GRP78 epitopes	Position
LLLLSAARAEE	AA10-20
VAFTPEGERLIGD	AA66-78
IESFYEGED	AA309-317
AGVLSGDQDTGD	AA402-412
PEEIERMVNDAEK	AA534-547
ESYAYSLKNQIGD	AA567-579

**Table 2:** Main GRP78 epitopes differentially recognized by GRP78 autoantibodies from serum compared to ascites

Peptides	Amino acids sequence	Sequence in GRP78
1	RIDTRNELESYAY	AA558-571
2	IDTRNELESYAYS	AA559-572
3	ITPSYVAFTPEGE	AA61-73
4	DQDTGDLVLLDVC	AA408-420
5	RIINEPTAAAIAY	AA197-209
6	ESYAYSLKNQIGD	AA567-579

### Epitope involved in decreased cell viability

**Figure 3:**
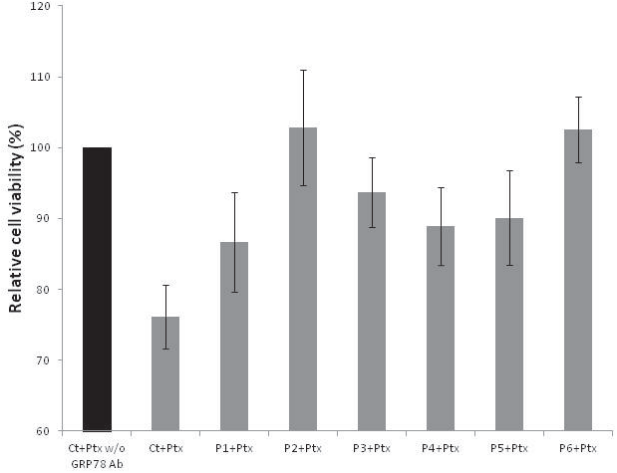
Relative viability of cells treated with paclitaxel, with or without GRP78 Ab and peptides 1 to 6

Considering that GRP78 antibodies purified from ascites and serum affect cells in different ways, we treated cells with the peptides corresponding to the epitopes that had been differentially recognized by anti-GRP78 antibodies in ascites or serum. This treatment was done in the presence and absence of GRP78 autoantibodies (purified from serum) as well as Ptx. This competition study revealed that at least one epitope contained in the sequence AA559-579 was involved in decreasing cell viability (Figure 3).

We thus decided to use the commercial polyclonal antibody LS-C165063 recognizing the epitope aa548-587 to coat Ptx-loaded NPs.

### GRP78 antibody-coated nanoparticles

#### Characterization

**Figure 4:**
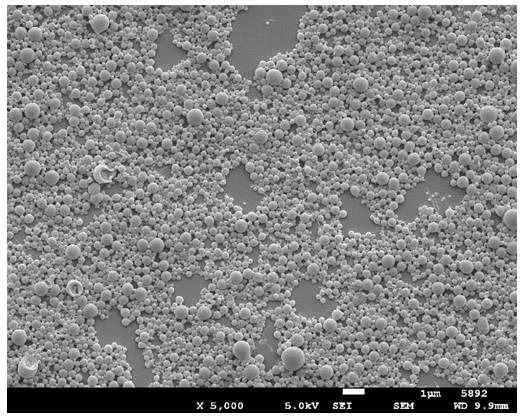
Scanning electron microscopy picture of nanoparticles Magnification: X5000; bar=1μm.

The NP characteristics are summarized in Table 3. The SEM pictures display spherical and smooth particles (Figure 4). The nanoparticles were 380 nm in size with a 0.2 polydispersity index. The nanoparticles were loaded with 80 μg/mg of Ptx.

**Table 3:** Physicochemical characterization of NPs

	Size nm	Polydispersity index	Paclitaxel loading (mg/mg)	Ab/NP (μg/mg)
NP-Ptx	351	0.15	83.1	-
NP-Ptx-Ab	380	0.21	80.1	13.5

#### Internalization

**Figure 5:**
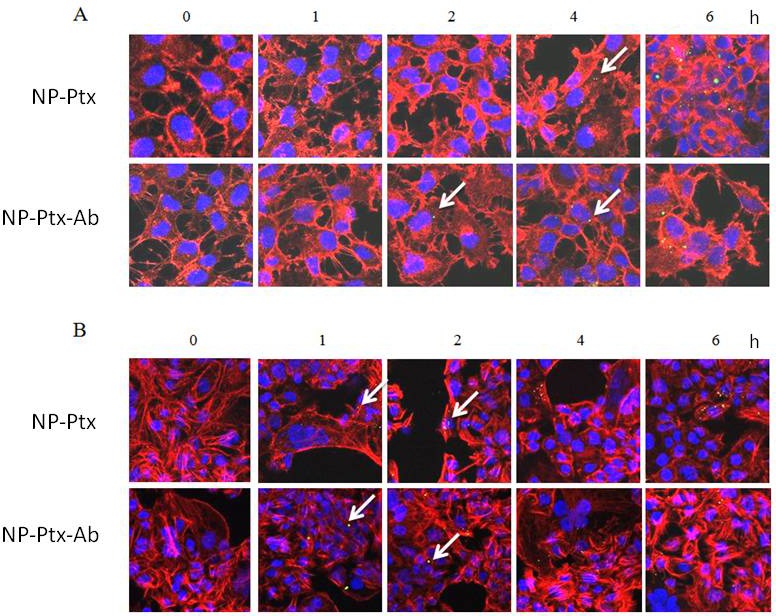
Internalization of nanoparticles in SKOV3 **(A)** and COV318 **(B)** cells. White arrows show internalized nanoparticles. Green staining: DiO labeled NP; Red staining: Phalloidin. Magnification: X400**.**

SKOV3 cells and COV318 cells were treated with NP-Ptx or NP-Ptx-Ab fluorescently tagged with DiO for 6 h, 4 h, 2 h, and 1 h and then fixed and stained with rhodamine-phalloidin for immunofluorescence imaging. In both treatments (NP-Ptx and NP-Ptx-Ab) there is evidence of nanoparticles (green fluorescent dots) internalized into cells from 2 h for SKOV3 cells and 1 h for COV318 (Figure 5). Also, at the longer treatment time of 6 h more green NPs were associated with the cells than at 1 h. This is true for both NP-Ptx and NP-Ptx-Ab treatments. The untreated control cells had no evidence of DiO green staining, as expected.

#### *In vitro*: cell viability assay – cytotoxicity

**Figure 6:**
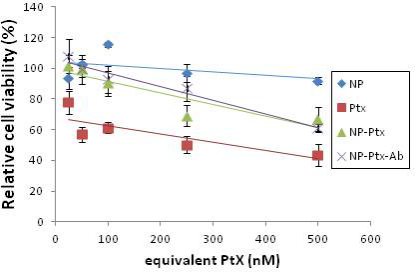
Cell viability of COV318 cells treated with unloaded NP, free Ptx, NP-Ptx or NP-Ptx-Ab COV318 cells were cultured for 48h with different formulations and concentrations of equivalent Ptx before MTT assay was performed.

COV318 cells were treated with NPs, Ptx, NP-Ptx, and NP-Ptx-Ab, and incubated for 48h, then assessed with a cell viability assay. NPs without Ptx were the control for the cytotoxicity of the polymer and did not influence cell viability (Figure 6). *In vitro*, the NP-Ptx had the same effect as the NP-Ptx-Ab. Free paclitaxel is much more efficient than NP-Ptx (Figure 6). These results were confirmed on SKOV3 cells (data not shown).

#### *In vivo*: chick embryo culture

**Figure 7:**
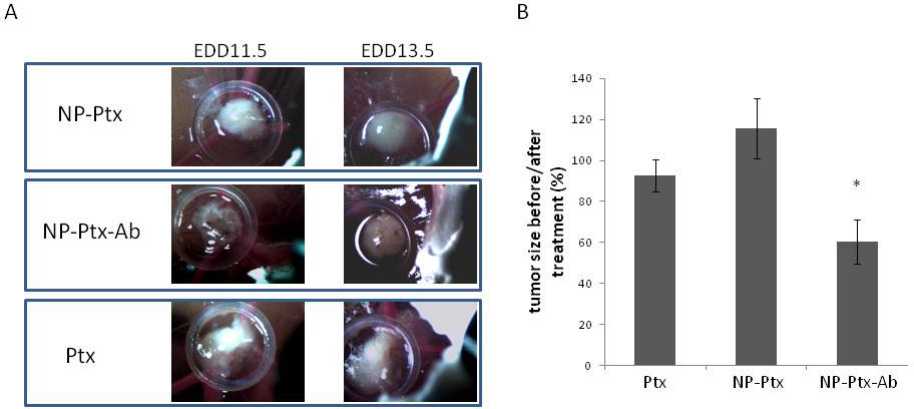
Effects of Ptx, NP-Ptx and NP-Ptx-Ab on tumor development Tumor size was monitored using a wild Hoerbrugg M3Z microscope at 10X magnification at EDD 11.5 (before treatment) and EDD13.5 (48h of treatment). The tumor size before and after treatment was determined using ImageJ Software.

The COV318 cells were inoculated into chick embryo culture and the tumors that developed were treated with Ptx, NP-Ptx, or NP-Ptx-Ab for 48 h. Tumor growth was compared between treatment day (T=0) and day 2 of treatment (T=2). These days correlate to embryonic development days (EDD) 11.5 and 13.5 respectively. The Ptx and NP-Ptx treatments had similar growth percentages, while NP-Ptx-Ab had approximatively 30% reduced growth compared to free Ptx. The reduced growth of the NP-Ptx-Ab treatment was statistically significant compared to the free Ptx (Figure 7).

## DISCUSSION

In an effort to increase the diagnostic specificity of cancers, biomarkers derived from the humoral immune response against tumor-derived proteins have shown promise. By studying the serum of prostate cancer patients, several peptides were identified based on immunoglobulin binding. There was an increase in reactivity against GRP78 as prostate cancer progressed, however when tested in other cancer patients, specifically advanced ovarian cancer, there was significantly less reactivity.

A diagnostic array utilizing exosome-derived antigens was used to detect immunoglobulins in ovarian cancer patient sera. For control and benign disease, samples were negative to all the antigens on the dot-blot array. Differentiation between benign and malignant ovarian tumors was based on autoantibodies against nuclophosmin, GRP78, cathepsin D, and SSX. Specifically, the study indicated antibodies against GRP78 were greater in the later stages of ovarian cancer compared to control and benign tumors [5]. However, in an ELISA detecting the presence of GRP78 autoantibodies, the level was lower for ovarian cancer compared to the benign disease [15]. Here, we confirmed the presence of GRP78 at the surface of ovarian cancer cells and the potent role of membrane GRP78 in cell proliferation by demonstrating a positive correlation between membrane GRP78 expression and relative proliferation of cells. Despite the fact that the membrane GRP78 level is increased in ovarian cancer cells, we found that the mean level of circulating GRP78 autoantibodies was lower, although not significantly, in serum of ovarian cancer patients compared to controls (Figure 2A). This result is similar to the results reported by Mintz et al. [16]. Thus, the level of circulating GRP78 autoantibodies did not correlate with the presence of GRP78 at the cell surface in ovarian tumor cells (Figure 2B). We also described that ovarian cancer patient sera contain different GRP78 antibodies, recognizing different GRP78 epitopes. This observation and the discrepancies in literature suggest the need for a more specific ELISA to obtain accurate GRP78 autoantibody quantification in serum of patients, as it was described by Selim et al. for melanoma patients [20]. This study indicates that only a subpopulation of autoantibodies triggers melanoma cell proliferation. This type of assay should be applied in the ovarian cancer patient population.

As expected, malignant ascites also contains different GRP78 autoantibodies. Most of them are similar to those found in serum; however, their relative proportion seems to be different in these two fluids. B1 cells may produce variant antibodies in the peritoneal cavity. We could also expect diffusion in serum and vice versa, leading to very similar if not identical antibody populations. Our results indicate that this is not exactly the case. Considering that GRP78 autoantibodies purified from ascites and serum do not affect cells in similar way, this difference allowed us to define the GRP78 epitope involved in pro-apoptotic properties.

Ovarian cancer is the deadliest of all gynecological tumors, and with a five-year survival rate under 50%, [1] it makes for a difficult cancer to fight. New drug delivery systems are being studied to combat cancers. Specifically, NPs are proving to be effective in the transport of drugs to cancer cells [21–23]. NPs at a size of 100-400 nm can be loaded with poorly water soluble drugs, such as Ptx, as a more effective way for drugs to travel through the body. Additionally, NPs can be coupled with ligands, such as antibodies, for an active targeting approach. The antibodies on NPs recognize and bind to the biomarkers specifically overexpressed on cancer cells, helping to reduce the adverse effects on normal cells. Previous studies have shown that Ptx encapsulated NPs with surface coupling improved anticancer activity in disseminated xenograft ovarian cancer and increased bio-distribution at the target site [24]. It’s been shown that by using the cell surface expression of GRP78 as a target in ovarian cancer cells, antibodies against the C-terminus of GRP78 coupled to NPs loaded with Ptx can enhance the cytotoxicity of the drug *in vitro* [21]. The purpose of this study was to test Ptx-loaded NPs coupled with anti-GRP78 antibodies identified in serum of ovarian cancer patients for apoptotic activity on cancer cells *in vitro* and *in vivo*. Polylactic acid (PLA) polymer was chosen for the matrix of the particles because of its well-known biocompatibility when used in implants or microspheres for injectable slow release delivery systems. These have been approved by the FDA and numerous formulations are already on the market, for example Nutropin Depot^®^, Decapeptyl^®^, Trelstar^™^ Depot as microparticles or Zoladex^®^, as implants. Another important aspect is the physio-chemical properties of the polymer. Indeed, as a hydrophobic polymer it has high affinity with a drug such as paclitaxel, allowing high encapsulation rate. Finally, non-end-capped PLA allows for the coupling with entities such as antibodies. When considering GRP78 as a target, other polymers have been used. Albumin was used to encapsulate the more hydrophilic 5-FU by Zhao et al [25] in which the particles were coated with a monoclonal antibody against GRP78. Passerela et al. encapsulated paclitaxel in another polyester recently developed (poly(valerolactone-epoxyvalerolactone)) to target breast cancer cells and glioma cells with a linked peptide using the click chemistry [26]. However, this is a new polymer, meaning a long developing time will be necessary.

The ability for NPs to internalize into SKOV3 and COV318 cancer cells was demonstrated using immunofluorescence imaging. Ptx loaded NPs (NP-Ptx) and antibody coupled Ptx-loaded NPs (NP-Ptx-Ab) were tested and there was evidence of internalization for both treatments.

*In vitro*, in COV318, free Ptx was more efficient than nanoparticles whether they were coated or not. As with nanoparticles, Ptx needs to be released from the matrix because with an incubation time of 24h, it is possible that not all the drug content was freed from the particles to be available for cytotoxic activity. However, this is in disagreement with Cirstoiu et al. [24], in which the incubation schedule was different and it may be explained by a larger diffusion of the drug from the nanoparticles.

The targeted drug therapy approach was effective against ovarian cancer supported by the NP-Ptx-Ab decreased tumor growth in chick embryo compared to Ptx treatment alone and Ptx-loaded-NPs (Figure 7). By presenting the Ptx in a targeted nanocarrier, it induced cell penetration of Ptx as a chemotoxic agent. This hypothesis is in agreement with Zhao et al. who demonstrated the superior efficiency of Ab-coated 5-FU loaded-NP in hepatocarcinoma cells [25]. However, when tested *In Vitro*, the cytotoxic effects of NP-Ptx and NP-Tx-Ab were lower than free Ptx. To explain these differences between *in vitro* and *in vivo* results, we hypothesize that there is a better drug distribution with more stability of the GRP78 Ab coated NPs compared to the free Ptx or NP-Ptx *in vivo*. Since the antibody coupled Ptx loaded NPs treatment showed favorable results in decreasing tumor growth in CAM, the next step will be to test the treatment in mice to further understand the effects on tumor growth.

## MATERIALS AND METHODS

### Materials

Poly (DL-lactic acid) (100DL 4A, Mw 57kDa) was provided by Lakeshore Biomaterials, Inc (Birmingham, AL). 1-Ethyl-3-(3-dimethylaminopropyl)-carbodiimide (EDAC), D(+)-Trehalose dihydrat, Phosphate buffer saline (PBS), Poly-L-Lysine solution, 0.1% (w/v) from Sigma (Buchs, Switzerland), m-maleimidobenzoyl-N-hydroxy-sulfosuccinimide ester (sulfo-MBS), Tris (2-carboxyethyl)-phosphine hydrochloride (TCEP) and D-salt dextran plastic columns were supplied by Pierce (Rockford, IL USA), Dioctadecyloxacarbo-cyanine perchlorate (DiO) was from Molecular Probes (Leiden, The Netherlands). Poly(vinyl alcohol) (Mowiol 4–88) was purchased from Hoechst (Frankfurt/M, Germany). Paclitaxel (Ptx) was obtained from Cfm Oskar Tropitzsch (Marktredwitz, Germany). GRP78 peptides were synthesized by PEPperPRINT (Heidelberg, Germany).

### Serum

The study was approved by the ethics committee of the Geneva University Hospital and a written informed consent was obtained from patients. Sera were obtained from 71 control and 31 HGSOC patients. No significant differences were identified in the mean age between the CTRL and HGSOC groups.

### Primary ovarian cells

The study was approved by the ethics committee of the Geneva University Hospital and a written informed consent was obtained from patients. Ovarian cells were purified from ovarian tissue or ascites as described previously [3].

Cells were cultured in DMEM medium supplemented with 10% FBS and gentamicin at 37°C in a humidified incubator containing 5% CO_2_. They were characterized by PCR (CD90, HE4, PAX8, cytokeratin 8, cytokeratin 19, cytovillin) and by immunocytochemistry (cytokeratin 7, cytokeratin 18, cytokeratin 19, vimentin, p53) and used before passage 5.

### Cell-ELISA

Purified ovarian cells were seeded at and 3 × 10^4^ cells/well in a 96-well plate. Cells were either incubated directly with the primary antibodies or washed, fixed (3% PFA in PBS), washed, permeabilized (0.2% triton in PBS) and pre-incubated with culture medium (30 minutes) before incubation with anti-GRP78 antibodies (Sigma Aldrich, St Louis, MO, USA) for 45 minutes (dilution: 1/200). To remove the unbound antibodies, cells were washed four times in culture medium, and then incubated 30 minutes at 4°C with HRP conjugated goat anti-rabbit IgG antibody (dilution: 1/500). After incubation, cells were washed as described above and the substrate 3, 3′, 5, 5′- tertramethyl benzidine (MTT) (R&D systems, Minneapolis, USA) was added. The reaction was stopped by adding 0.2 M sulphuric acid. Absorbance was read at 450 nm on a microplate reader. These experiments were carried out in triplicate, two times (at two different passages).

### Proliferation assay

SKOV3 cells (as reference) and primary ovarian cells were seeded in 96-well plates at a density of 30’000 cells/well and incubated for 48h. Culture medium was then replaced by 100μl of MTT reagent. Plates were analyzed after 2 hours using a microplate reader at λ=540 and 690 nm (as blank). Cell viability was calculated by comparing the absorbance of primary ovarian cells with the absorbance of SKOV3 cells. These experiments were carried out in triplicate, two times (at two different passages).

### Anti-GRP78 ELISA

Anti-GRP78 ELISA was realized as previously described [27].

### Epitope mapping

Epitope mapping of ascites and serum obtained from the same patient against GRP78 was realized by PEPperPRINT (Heidelberg, Germany)[28]. Briefly, the protein sequence of GRP78 was translated into 13mer peptides with a shift of one amino acid. The C- and N-termini were elongated by neutral GSGSGSG linkers to avoid truncated peptides. The peptides were spotted on a microarray and incubated with the two fluids (diluted 1:100) at 4°C overnight. Peptide microarrays were stained with fluorescence-labeled secondary antibodies and read by an Imaging System (Odyssey Imaging, USA).

### Ptx-loaded immunoparticle preparation

Ptx-loaded NPs were prepared by a salting-out process as described previously [21]. The same method was used for fluorescently labeled NPs, Ptx was then replaced with 3,3-dioctadecyloxacarbo-cyanine perchlorate (DiO) (0.01% w/w).

Grafting of the anti-GRP78 antibody (LS-C165063, LSBio, LabForce AG, Muttenz, Switzerland) was obtained using a carbodiimide method as already described [21]. In the first step, the free carboxyl groups of the polymer at the NPs surface were thiolated (-SH), then there was covalent attachment of mAbs to thiolated NPs via a sulfo-MBS (m-maleimidobenzoyl-N-hydroxy-sulfosuccinimide ester) cross-linker. Briefly, PLA NPs were suspended into water and reaction initiated by adding, consecutively a solution of EDAC (1-Ethyl-3-(3-dimethylaminopropyl)-carbodiimidide) then cysteine solution. The final suspension was stirred under mild conditions for 24h at room temperature. Remaining EDAC and non-reacted cysteine were removed by centrifugation. The NPs were then re-suspended in purified water before reduction of disulfide bonds by reaction of a solution of TCEP (Tris (2-carboxyethyl)-phosphine hydrochloride). Finally, the NP-SH were purified by centrifugation and freeze-dried in presence of trehalose to avoid aggregation. In the following step, the anti-GRP78 Ab was activated in phosphate buffer saline (PBS, pH 7.4) with sulfo-MBS. After removing non-reacted sulfo-MBS by size exclusion chromatography using desalting columns, the activated ligand was incubated with NP-SH and gently shaken at room temperature. Unconjugated ligand was removed by centrifugation.

### Characterization of NPs

The size and morphology of nanoparticles were analyzed by photon correlation spectroscopy (PCS) using a Zetasizer 3000 HS (Malvern instruments Ltd, UK), and scanning electron microscopy (SEM) performed on gold-coated freeze-dried samples (Balzers SCD 004 Sputter Coater) with a JEOL JSM-6400 microscope (JEOL, Tokyo, Japan) at an accelerating voltage of 10 or 15 kV.

The drug loading was determined by HPLC method after dissolution of the polymeric matrix [18]. Three milligrams of NP-Tx were dispersed in 1 mL of acetone and sonicated for 5 minutes. The solution was centrifuged (Beckman Avanti™ 30 centrifuge, rotor 1202). Paclitaxel content in supernatant was measured by reverse-phase HPLC with UV detection. The HPLC system consisted of a Waters 600 Controller separation module fitted with a Waters 2487 Dual λ absorbance detector, a Waters 717 Plus Autosampler and a NUCLEOSIL C18 column, 4.6mm x 125m, 5 μm, (Macherey-Nagel, Switzerland). The mobile phase, (acetonitrile: water = 50:50), was delivered at a flow rate of 1ml/min, the volume of injected sample was 20 μl and the drug was detected at 227 nm. A standard plot for Tx (0.08-0.50 mg/ml) was prepared under identical conditions. The encapsulation efficiency (%) was calculated as percentage of drug entrapped into the NPs with respect to the initial amount of drug added in the formulation and the drug loading was expressed as the amount of drug encapsulated in 100 mg NPs.

The amount of mAb conjugated to nanoparticles was determined indirectly by measuring uncoupled mAb in the supernatant after the centrifugation step. A spectrophotometric method was used at λ=280 nm (NanoDrop 1000 spectrometer, Thermo Scientific, USA).

### Cell culture

The human ovarian carcinoma cell lines, COV318 and SKOV3, were cultured respectively in DMEM or RPMI medium supplemented with 10% FBS and gentamicin at 37°C in a humidified incubator containing 5% CO_2_.

### Immunofluorescence

SKOV3 and COV318 cells were plated at a density of 3 × 10^4^ cells in Lab-Tek^®^ II Chamber Slide™ (Nunc, NY, USA) and cultured for 24 h. Cells were then exposed to 1 mg/ml of NP-Ptx or NP-Ptx-b labeled with DiO in a CO_2_ incubator at 37°C for different time points. After 10 washes in PBS, cells were fixed with 3% PFA at room temperature for 20 min and rinsed 3x5 min in PBS before being incubated with 1% (v/v) rhodamine-phalloïdin in PBS for 20 min at room temperature. Cells were then rinsed with PBS and a slide was mounted with Vectashield^®^ and observed with an Axiocam microscope (Zeiss, Oberkochen, Germany).

### Cytotoxicity assay

*In vitro* cytotoxicity of different NPs formulations was tested on COV318 using MTT assay and compared with Ptx.

Ovarian cancer cells were seeded in 96-well plates at a density of 3 × 10^4^ cells/well and incubated for 24h before treatment. Cell culture medium was then replaced by 100 μl of the different formulations (0, 25, 50, 100, 250 and 500 nM equivalent Ptx) for 48h. Culture medium was then replaced by 100μl of MTT reagent. Plates were analyzed after 2 hours using a microplate reader at λ=540 and 690 nm. Cell viability was calculated by comparing the samples to cells incubated with normal culture medium as 100% survival rate.

### Tumor development and treatment on chick chorioallantoic membrane (CAM)

#### Chick embryo culture

Fertilized eggs (animal facility of the University of Geneva, Geneva, Switzerland) were incubated at 38°C with 80% relative humidity and periodic rotation. Rotation was stopped on egg development day (EDD) 4 and eggs were drilled at their narrow apex. The hole was closed with adhesive tape. Incubation was carried out until use.

#### Cells grafting

On EDD8 the hole in the eggshell was enlarged to allow the access to the CAM. After gently scratching of the membrane with a needle tip, a silicon O-ring (Apple Rubber products inc., Lancaster, USA) was placed onto a blood vessel crossing. COV318 cells suspension (2X10^6^ cells in 30 ul) were placed into the silicon O-ring and the hole was hermetically covered with Parafilm®. Eggs were returned to the incubator for 3 days to allow tumor growth. Tumor growth was monitored using a Wild Heerbrugg M3Z microscope at 10x magnification with a Lumenera INFINITY2-1 CDD camera with Infinity Capture Software.

#### Treatment

Three days after the grafting (EDD11), available tumors were treated topically with 100 μM equivalent Ptx. Eggs were returned to the incubator for 2 days (EDD13). Tumor size was monitored using a Wild Heerbrugg M3Z microscope at 10x magnification with a Lumenera INFINITY2-1 CDD camera with Infinity Capture Software.

### Statistical analysis

Results were measured and expressed as mean +/- SE. The differences between samples were evaluated by Student’s t test and p value < 0.05 was considered significant.

The Pearson correlation coefficients were calculated by comparing the level of membrane GRP78 with proliferation of cells or levels of circulating anti-GRP78 antibodies and p value < 0.05 was considered significant.

## SUPPLEMENTARY MATERIALS




