# Supplementary material for: Circulating GRP78 antibodies from ovarian cancer patients: a promising tool for cancer cell targeting drug delivery system?
Source: Oncotarget. 2017 Nov 11;8(63):107176–87. doi: 10.18632/oncotarget.22412 (PMC5739806; doi:10.18632/oncotarget.22412)
Supplement: Supplementary file 1 [file oncotarget-08-107176-s001.pdf]

Circulating GRP78 antibodies from ovarian cancer patients: a promising tool for cancer cell targeting drug delivery system?

SUPPLEMENTARY MATERIALS

Supplementary Data: Differential analysis of serum versus ascites peptide map

See Supplementary File 1
